# An open-source non-contact thermometer using low-cost electronic components

**DOI:** 10.1016/j.ohx.2021.e00183

**Published:** 2021-03-06

**Authors:** Mohannad Jabbar Mnati, Raad Farhood Chisab, Azhar M. Al-Rawi, Adnan Hussein Ali, Alex Van den Bossche

**Affiliations:** a Institute of Technology Baghdad, Middle Technical University, Baghdad, Iraq; b Technical Institute Kut, Middle Technical University (MTU), Baghdad, Iraq; c Electrical Power Techniques Department, Al-Mamon University College, Baghdad, Iraq; d Department of Electrical Energy, Metals, Mechanical Constructions and Systems Ghent University, Ghent, Belgium

**Keywords:** Non-contact thermometer, GY-906 MLX90614ESF, Arduino UNO, TCRT 5000, 16*2 LCD, DS3231, SD storage board

## Abstract

Due to the spread of COVID-19 across the world and the increased need for non-contact thermometers to prevent the spread of disease, a new electronic thermometer has been designed and implemented for measuring human body temperature from a distance. This device is currently in use at building entrances to measure the body temperatures of employees, students, and customers. This system is designed using low-cost easy-to-assemble open-source electronic components. The system consists of seven main parts: an Arduino UNO microcontroller, an infrared (IR) thermometer for non-contact temperature measurements (GY-906 MLX90614ESF module), an IR motion sensor (TCRT 5000) for the purpose of contactless initiation of the system, a graphic LCD to display results, a DS3231 clock module for a real-time clock and calendar, and a micro-SD storage board to store device audio instructions.

## Graphical abstract

**Figure:**
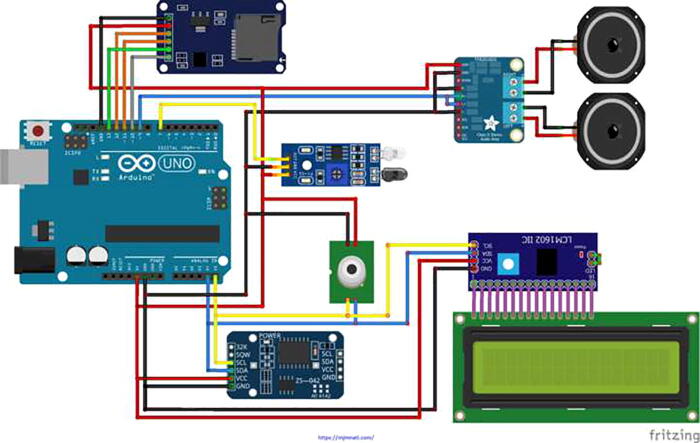

Specifications table:

**Table:** 

Hardware name	*Non-contact thermometer*
Subject area	• Engineering and Material Science • Medical (e.g., Pharmaceutical Science) • Educational Tools and Open-Source Alternatives to Existing Infrastructure • General
Hardware type	• Measuring physical properties and in-lab sensors • Field measurements and sensors • Electrical engineering and computer science
Open-Source License	GNU General Public License (GPL)
Cost of Hardware	$73 USD
Source File Repository	https://osf.io/w4kqs/files/

## 1 Hardware in context

A variety of methods for measuring a human’s body temperature exists. Body temperature has historically been measured using contact thermometers either mounted on the forehead or inserted into the mouth, ear, armpit, or rectum. Rectal temperature measurement is considered the gold standard, particularly for children. Non-contact thermometers enable an individual’s temperature to be taken with either limited (tympanic) or no contact (non-contact infrared thermometer, thermal scanner). This means that temperature can be determined without the pain of having to keep the mouth, armpit, or rectum immobilized with a thermometer long enough to get an accurate reading. Precluding the need for contact often means that thermometer disinfection between patients is unnecessary, allowing for faster use when screening large numbers of people in environments such as ministries, universities, and government departments [1], [2], [3], [4], [5].

There are many types of non-contact thermometers available, but we designed a new, low-cost, and easy to use non-contact infrared thermometer for this paper. Our device is designed to be placed near the main entrances to institutions and departments to measure people’s temperatures and present the results on a 16*2 LCD. After that, entry is allowed or denied because this device can issue instructions in either English or Arabic, without human control.

## 2 Hardware description

**Fig. 1:**
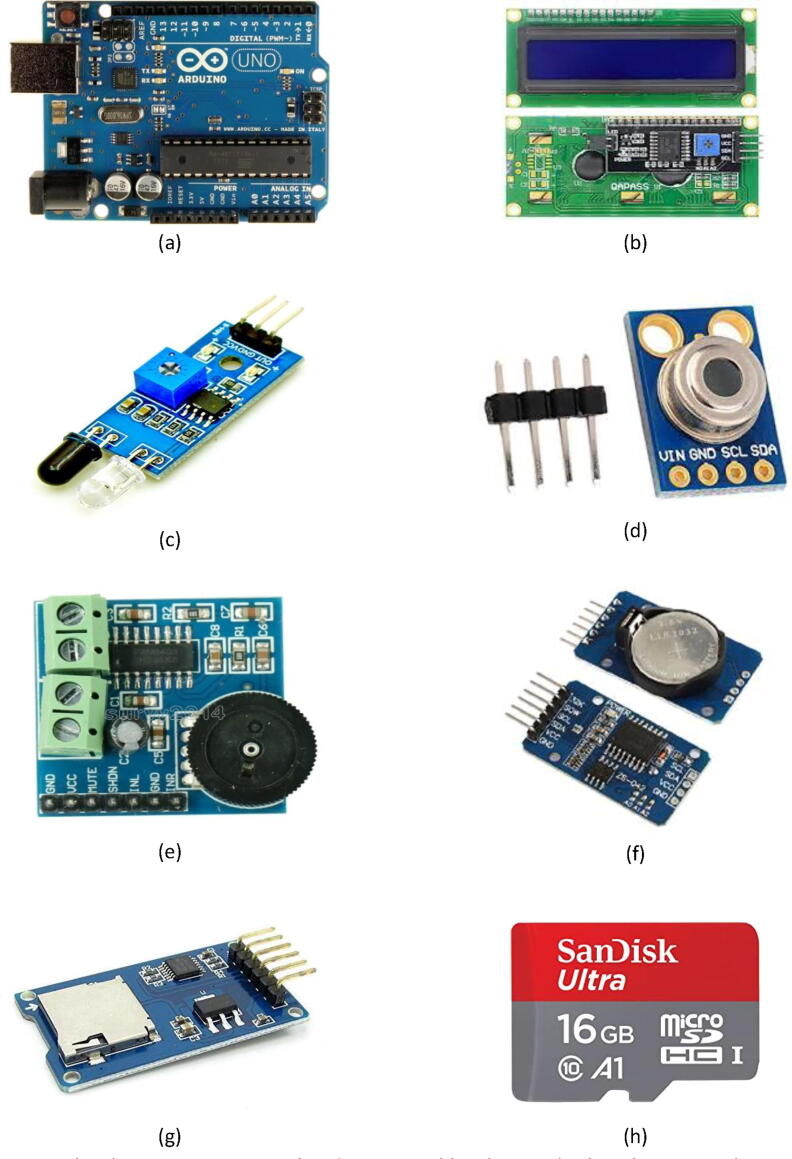
System hardware components: a) Arduino UNO, b) 16*2 LCD display, c) IR Sensor (TCRT5000L module), d) MLX90614 infrared thermometer, e) digital power amplifier (PAM8403 module), f) real-time clock (DS3231 Module), g) micro-SD storage expansion board, and h) 16 GB SD card.
2- 16*2 LCD display module: This alphanumeric LCD unit, which means that it can display letters of the alphabet and numbers. It consists of two rows; each row can print 16 characters (each letter is created by a rectangle of 5 × 8 pixels). It can operate in both 8-bit and/or 4-bit modes. The DC operating voltage is 4.7 V to 5.3 V without backlight, and the current consumption of the LCD is 1 mA. Typical LCDs usually need about 12 connections, which can be problematic if there are not many GPIO pins available. However, by using the I2C add-on circuit separately, we can easily upgrade the standard LCD, requiring only four GPIOs (two for power and two for data) [7], [8], [9] (see Fig. 1b).
3- IR sensor/TCRT5000L module: This sensor is an infrared-reflective sensor used for detecting lines and edges. It can also be used to locate obstacles within close range. These sensors transmit an infrared beam and then measure its reflection. This sensor comes with a pair of infrared emitters and receivers at the front of the panel. Any time an object blocks the infrared source, it reflects the infrared. The reflected light then strikes the receiver, and the signal passes through an onboard comparator circuit. In addition, it will output logic LOW at the output pin, and the green LED will light up to show the detection; depending on the threshold, that is being changed [10] (see Fig. 1c).
4- MLX90614 infrared thermometer: The MLX90614 is an infrared thermometer used for non-contact temperature measurements. Both its infrared-sensitive heat detector chip and its ASIC signal conditioning are integrated into the same TO-39 case. The MLX90614 includes a built-in low noise amplifier, 17-bit ADC, and a powerful DSP module, which allow it to achieve high precision and accuracy. The main advantages of this sensor are its small size, low cost, and ease of integration. Its temperature range spans −40 °C–125 °C for sensor temperature and −70 °C–380 °C for object temperature [11], [12] (see Fig. 1d).
5- Digital power amplifier module: The PAM8403 is a class-D 3 W audio amplifier, high-definition sound. It provides low THD + N, allowing high-quality sound reproduction. Its recently developed filter-less architecture allows the device to drive the speaker directly without the need for low-pass output filters, thereby saving on system costs and PCB zone [13] (see Fig. 1e).
6- Real-time clock module: The DS3231 module is a low cost, highly accurate real time clock capable of retaining hours, minutes, and seconds, as well as information of the day, month, and year. It also has a measured automatic leap year (also known as an intercalary year or bissextile year), which balances months of less than 31 days. The module can operate on either 3.3 V or 5 V, making it suitable for many of the platforms or microcontrollers in production. Battery input is 3 V, and the module can be powered by a standard CR2032 3 V battery for more than a year. The module uses the I2C Communication Protocol to send data to the board of Arduino [14], [15] (see Fig. 1f).
7- Micro SD storage expansion board: Data storage is one of the most important parts of any project of this type. There are several ways to store data depending on its type and size. SD and micro-SD cards are some of the most practical types of storage devices, and they are used in devices like cell phones, small computers, and microcontrollers [16], [17] (see Fig. 1g and 1 h).

The complete system consists of the following seven major parts, all of which are open-source, inexpensive electronic components:

1- Arduino UNO unit: The Arduino Uno is a microcontroller board designed based on ATmega328P. It has 14 pins for digital input and output (only 6 can be used for PWM outputs signals), six analog inputs, a 16 MHz ceramic resonator, a USB link, a power jack, an ICSP header, and a reset key. The Arduino UNO contains everything needed to operate the microcontroller; it simply needs to be connected to a power source device with a USB cable or powered with a DC-adapted power supply or battery to get started. If the Arduino UNO is tampered with, it can be easily changed as this chip is cheap [6] (see Fig. 1a).

All electronic components are placed in a plastic box (174*225*30 mm) with a white acrylic cover (see Fig. 7). The complete system has the following features:

• Remote temperature measurement without the human intervention.
• The device speaks either English or Arabic in addition to displaying results on the LCD screen.
• The hardware uses open-source sensor controllers (Arduino).
• It is easy to use and easy to produce.
• Low cost.

## 3 Design files

For this paper, all system design files are available and maintained in the source file repository of Open Science Framework.

**Design files summary**

**Table:** 

Design file name	File type	Open-source license	Location of the file
Arduino and LCD1602 Interfacing circuit	Figure (PNG)	CC BY-SA	Available with article (Fig. 2)
Arduino and RTC Clock Interfacing circuit	Figure (PNG)	CC BY-SA	Available with article (Fig. 3)
Arduino and SD Card and Voice Interfacing circuit	Figure (PNG)	CC BY-SA	Available with article (Fig. 4)
Arduino and MLX90614 Sensor Interfacing Circuit.	Figure (PNG)	CC BY-SA	Available with article (Fig. 5)
Arduino and IR Infrared Sensor Interfacing Circuit.	Figure (PNG)	CC BY-SA	Available with article (Fig. 6)
Full system Hardware	Figure (PNG)	CC BY-SA	Available with article (Fig. 7)
Arduino code to test LCD1602	.ino	CC BY-SA	Source File Repository
Arduino code to test RTC Clock	.ino	CC BY-SA	Source File Repository
Arduino code to Test SD Card and Voice	.ino	CC BY-SA	Source File Repository
Arduino code to test MLX90614 (Digital Non-Contact Infrared Thermometer sensor)	.ino	CC BY-SA	Source File Repository
Full system Arduino Code	.ino	CC BY-SA	Source File Repository

## 4 Bill of materials

**Fig. 2:**
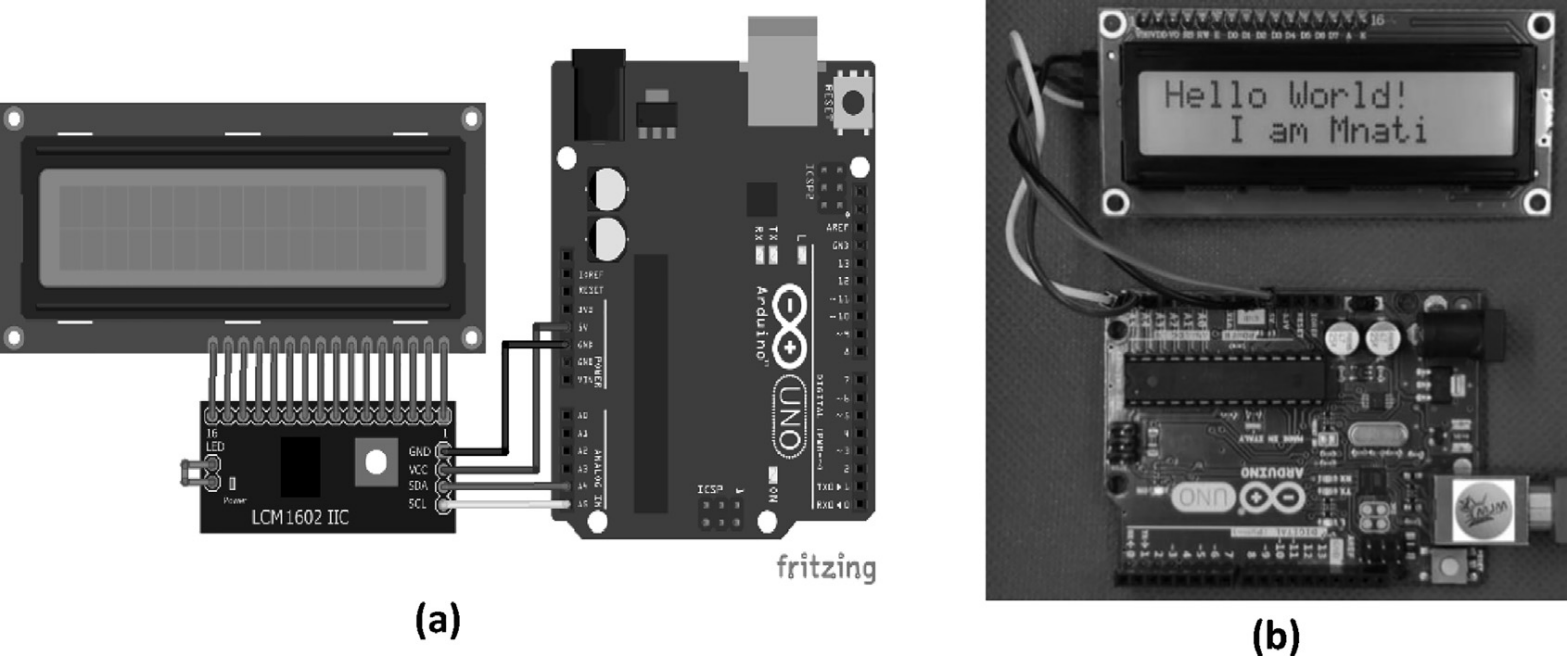
Arduino UNO and LCD1602 Interfacing Circuit: a) a schematic diagram of the circuit connections and b) the jumper wire connection between the Arduino UNO and the 16*2 LCD.

The cost of the electronic system components listed in Section 3 was calculated, considering the price difference between vendors. All prices are included in the following table. Fig. 2

**Fig. 3:**
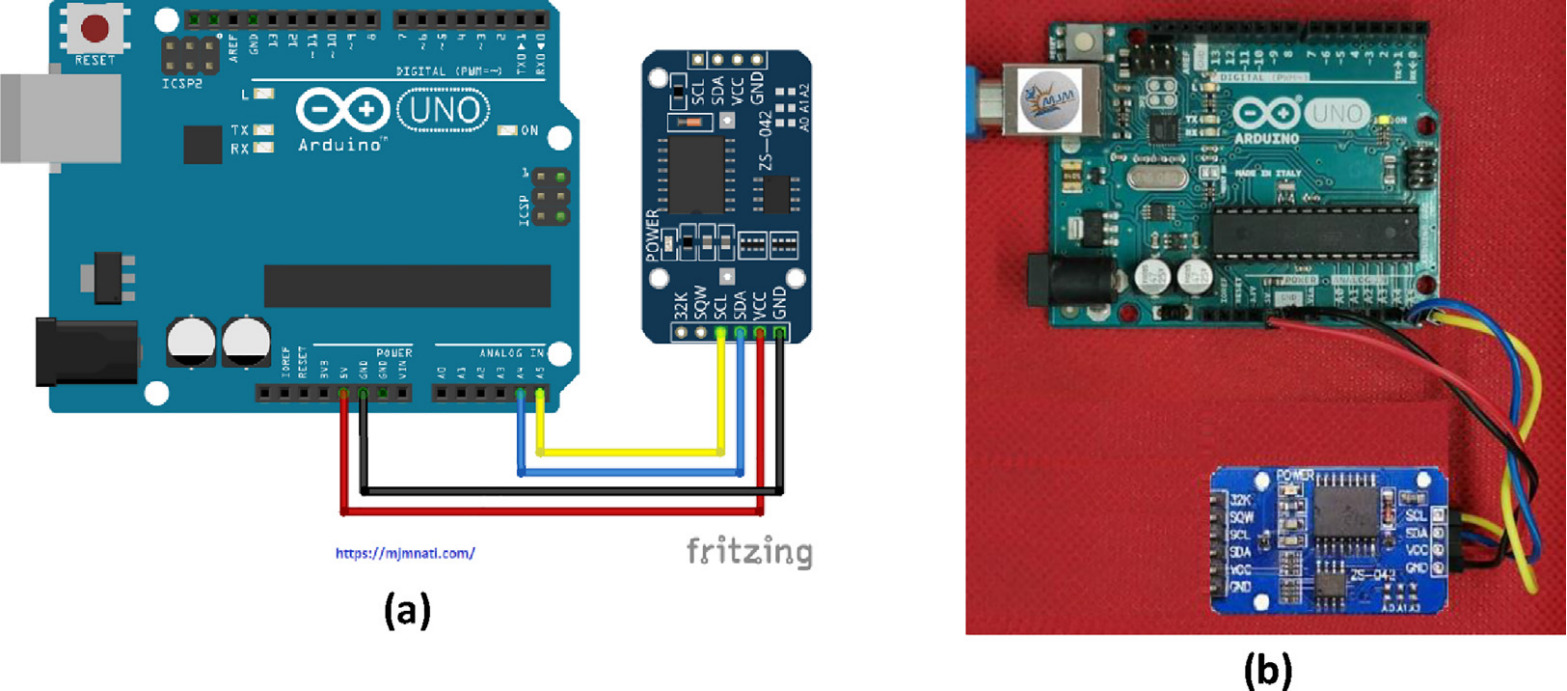
Arduino UNO and RTC Clock Interfacing Circuit: a) a schematic diagram of the circuit connections and b) The jumper wire connection between the Arduino UNO and the DS3231 module.

**Fig. 4:**
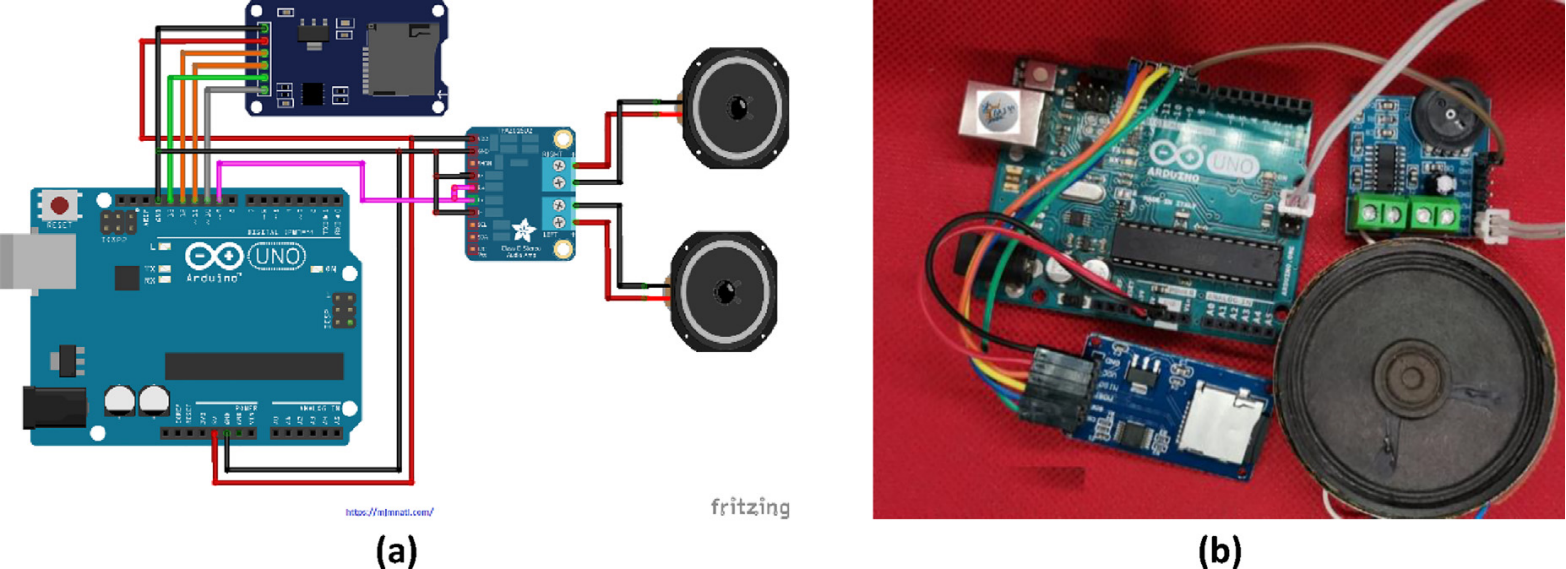
Arduino UNO, SD card, and voice interfacing circuit: a) a schematic diagram of the circuit connections and b) the jumper wire connections between the Arduino UNO, the SD card, and the amplifier circuit (mini pam8403a).

**Fig. 5:**
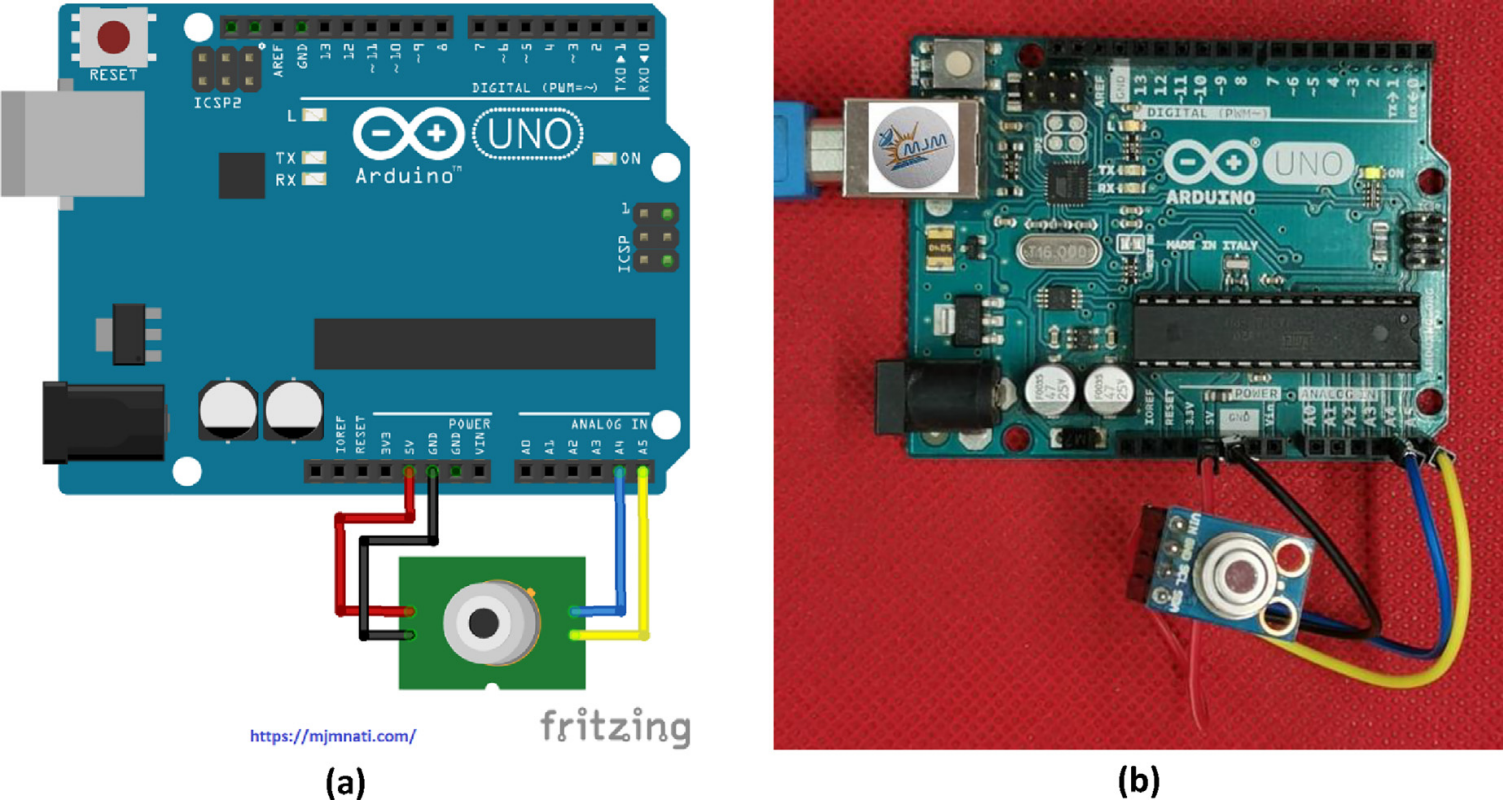
Arduino UNO and MLX90614 sensor interfacing circuit: a) a schematic diagram of the circuit connections and b) the jumper wire connections between the Arduino UNO and the MLX90614 sensor module.

**Fig. 6:**
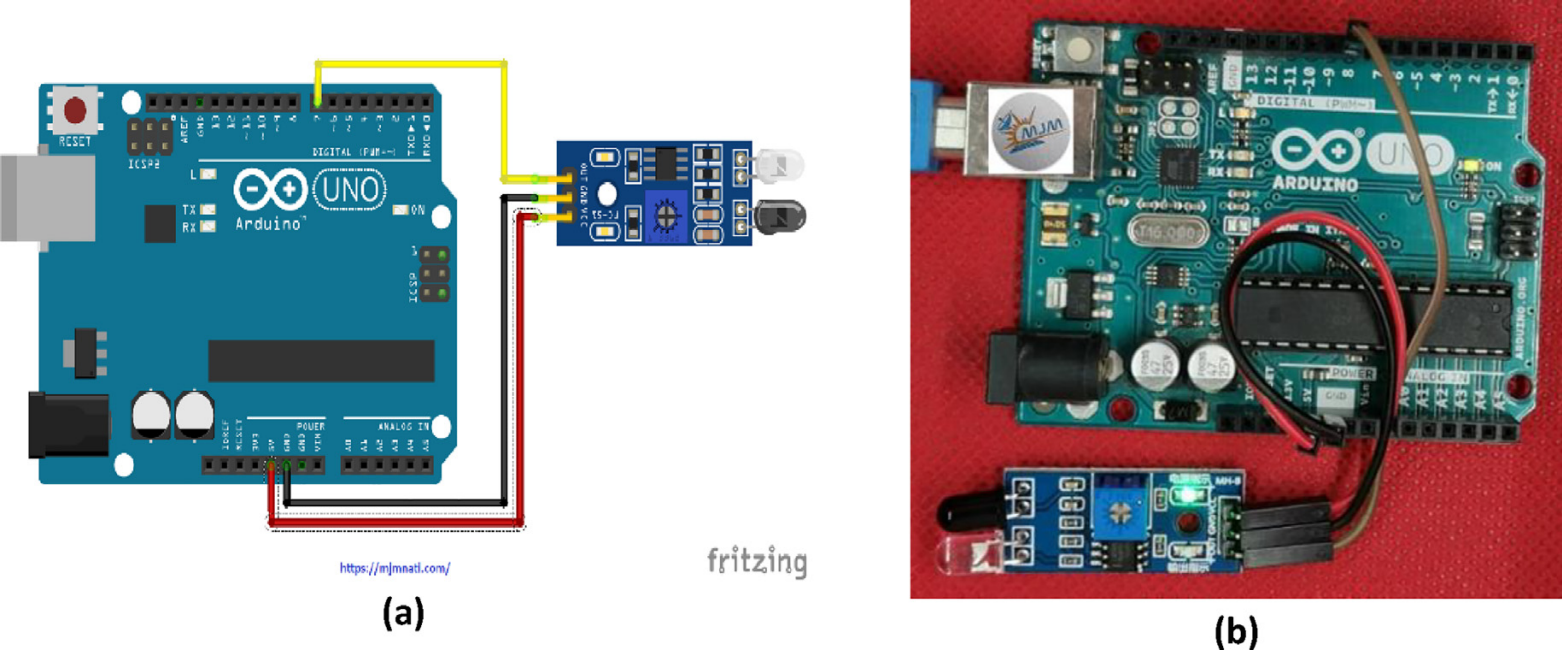
Arduino UNO and infrared sensor interfacing circuit: a) a schematic diagram of the circuit connections and b) the jumper wire connections between the Arduino UNO and the TCRT5000L module.

**Fig. 7:**
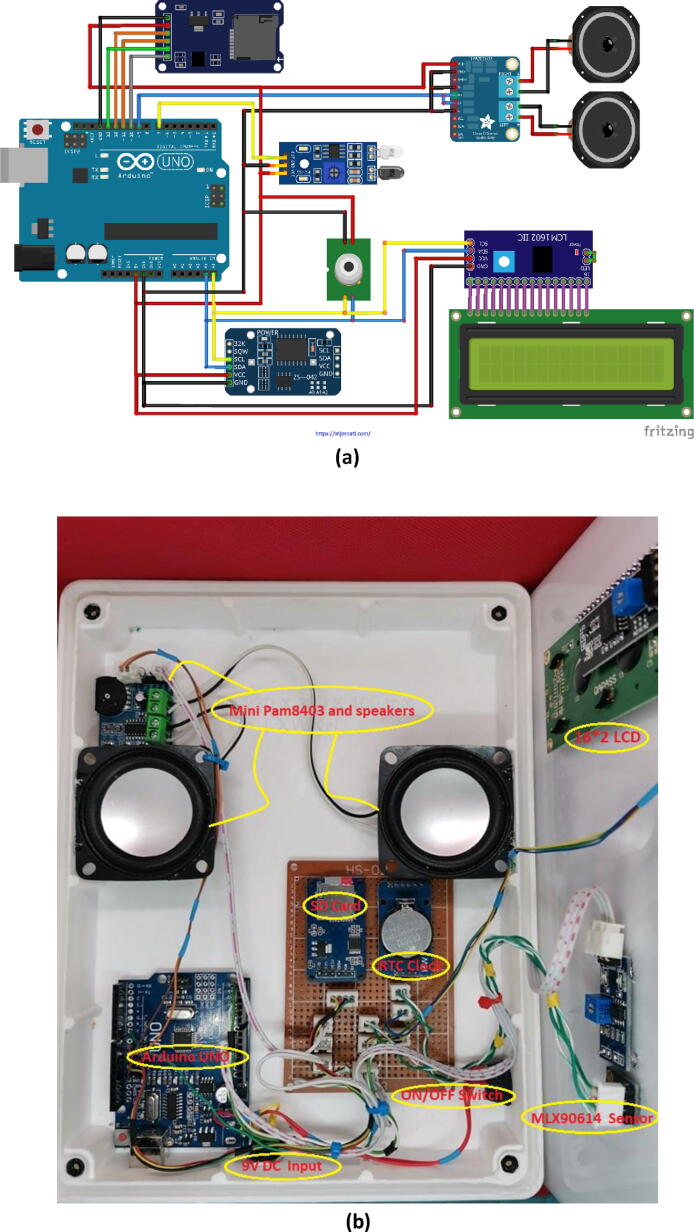
Full system Interfacing Circuit: a) Schematic diagram of the full circuit connections and b) Jumper wires connection between Arduino UNO and modules.

**Bill of materials**

**Table:** 

Component	Qty.	Unit Cost	Total cost	Source of materials	Material type
Arduino UNO	1	$4.60	$4.60	https://www.aliexpress.com/item/32831857729.html	Electronics
16*2 LCD with I2C	1	$1.78	$1.78	https://www.aliexpress.com/item/33052019740.html	Electronics
IR Sensor (TCRT5000L)	1	$0.36	$0.36	https://www.aliexpress.com/item/32321964595.html	Electronics
MLX90614 thermometer	1	$9.50	$9.50	https://www.aliexpress.com/item/32861472005.html	Electronics
Digital Power Amplifier	1	$0.48	$0.48	https://www.aliexpress.com/item/1822706737.html	Electronics
Real-Time Clock Module	1	$1.20	$1.20	https://www.aliexpress.com/item/1005001621707646.html	Electronics
Micro SD Expansion Board	1	$0.66	$0.66	https://www.aliexpress.com/item/32877331401.html	Electronics
Mini SD memory, 16 Gb	1	$3.52	$3.52	https://www.aliexpress.com/item/4000423662322.html	Electronics
Speaker	2	$3.73	$7.46	https://www.aliexpress.com/item/33038522252.html	Electronics
Power On/Off Switch	1	$0.99	$0.99	https://www.aliexpress.com/item/4001190014176.html	Electronics
Power Supply Connector	1	$0.85	$0.85	https://www.aliexpress.com/item/32883658107.html	Electronics
PCB connector cables	1	$3.32	$3.32	https://www.aliexpress.com/item/4000029865283.html	Electronics
Wires (deferent length)	2	$0.57	$1.14	https://www.aliexpress.com/item/32969968416.html	Electronics
Electrical Junction Box	1	$4.0	$4.0	https://www.aliexpress.com/item/4000287507400.html	Plastic
Acrylic white cover	1	$7.9	$7.9	https://www.aliexpress.com/item/1005001490026271.html	Acrylic
9 V DC power adapter	1	$3.0	$3.0	https://www.aliexpress.com/item/32961533195.html	
		Total	$50.76		
	Tax. + Shipping		$25.0		

## 5 Build instructions

Before the system can be completely designed and developed as a prototype, the electronic components should be tested separately. In this section, electronic circuit diagrams will be used to illustrate the practical part of each electronic component separately. It shows how these components work. In addition to that, it also shows how to upload the special program required to run each part of these components. Finally, the final design is provided with some examples of how the new device operate.

### 5.1 Arduino and LCD1602 Interfacing circuit

The first part of this section discuses the basics of presenting the text and numbers, and how to use the other features of the Arduino’s Liquid-Crystal library. This part includes everything that one must know about interfacing with and controlling LCD characters with Arduino. Fig. 3. presents wiring diagrams along with practical examples of the circuits. These displays are great for displaying sensor data or text, and they are cheap. The LCD was used with the I2C module mounted connected on the back. With this I2C module, only two connectors are needed to control the data on the LCD screen.

### 5.2 Arduino and RTC clock Interfacing circuit

The real-time clock module (DS3231) is the one that measures the time through its cell, either depending on or independent of its Arduino board. The module is a low cost, highly accurate real time clock capable of keeping hours, minutes, and seconds as well as data from day, month, and year. It also has automatic leap-year calculations and can set months that have less than 31 days. The Arduino card measures the time elapsed since the module was powered on (ms). The module is ready-to-use on installation and is supplied with a battery. The (DS3231) module used I2C protocol for the communication. This contact means that it interacts with the Arduino using only two pins.

### 5.3 Arduino, SD Card, and Voice Interfacing circuit

Fig. 4 illustrates how to play (.wav) audio files using the Arduino UNO board. The Arduino loads WAV files from a FAT16- or FAT32-formatted SD card; a mini pam8403a and speaker is then used to amplify the audio signal generated by the Arduino microcontroller. WAV is a Microsoft and IBM audio file format that is used as the standard medium for storing audio bit stream on PCs. A positive aspect of this format is that it is uncompressed, which allows even a small microcontroller to play it.

### 5.4 Arduino and MLX90614 sensor Interfacing circuit

Most temperature measurement apparatuses require some level of physical contact between the sensor and the object or environment whose temperature is being measured. However, as technology advances, this is no longer the case. When the need to measure body temperature without contact with the body arose, infrared sensors were used to meet this need.

The operating theory of infrared thermometers is very simple. All objects at temperatures above 0 K (absolute zero) emit infrared energy that can be measured by an infrared thermometer's sensor, the nature of which involves a lens that focuses on the detector’s infrared energy emitted by an object. The detector converts the energy into an electrical signal that can then be passed on to a microcontroller that interprets and displays it in units of temperature after compensating for the difference in ambient temperature.

The infrared thermometer’s rating plate is equipped with an easy-to-use, yet powerful, MLX90614 infrared thermometer that can sense temperatures between −70 °C and 380 °C. Using SMBus—a I2C-like interface—to communicate with the chip means that only two wires need to be dedicated from the microcontroller to interact with it.

### 5.5 Arduino and infrared sensor Interfacing circuit

The sensor device is adaptable to ambient light and comprises a couple of tubes that emit and receive infrared. The transmitting tubes emit infrared rays of a certain frequency; the receiving tube receives the reflected infrared rays when the obstacle (reflective surface) is detected. The green light comes on after the processing of the comparator circuit, but the output of the signal output interface is a digital signal (i.e. a low-level signal). The detection distance may be set on the handle’s voltage meter ranging from 2 cm to 30 cm, and the working DC voltage ranges from 3.3 V to 5 V. The sensor detection range can be obtained by changing the potentiometer with little interference. The unit is easy to assemble and use. It is widely applicable, being used to prevent obstacles to the sensor.

### 5.6 Final design of the system and results

After we learned how to use all the electronic parts used in our project individually, they were assembled in a plastic box and we wrote a single program to operate them together to serve the required purpose of this device. Fig. 7 presents the schematic diagram of the full system’s circuit connections and the jumper wire connections between all the electronic modules and the Arduino.

**Fig. 8:**
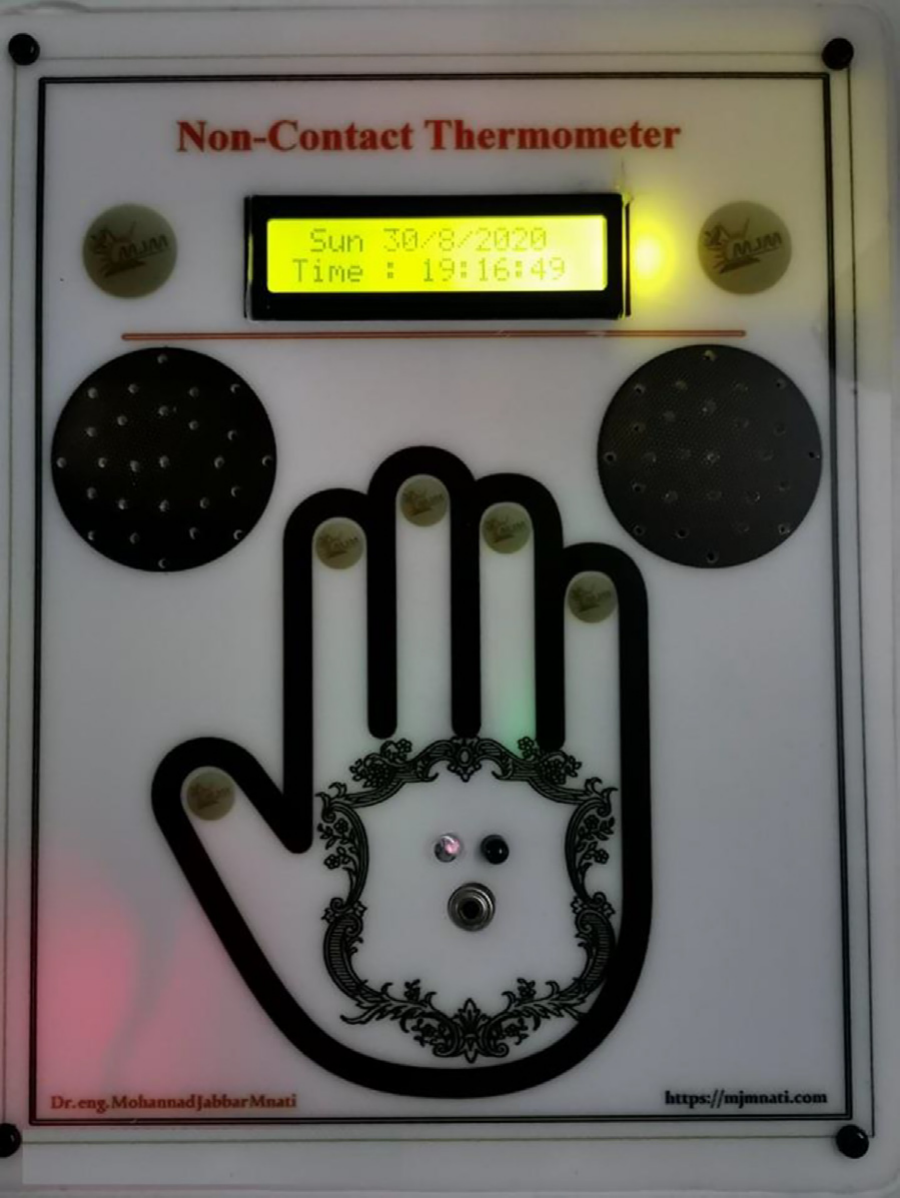
Front view of the full system under “working state” without measuring any temperature.

Fig. 8 shows the main interface (front view) of the prototype device after the final design was printed on a piece of white acrylic. Fig. 8b presents the device in a “working state” while not measuring any temperature (only the time and date are displayed).

**Fig. 9:**
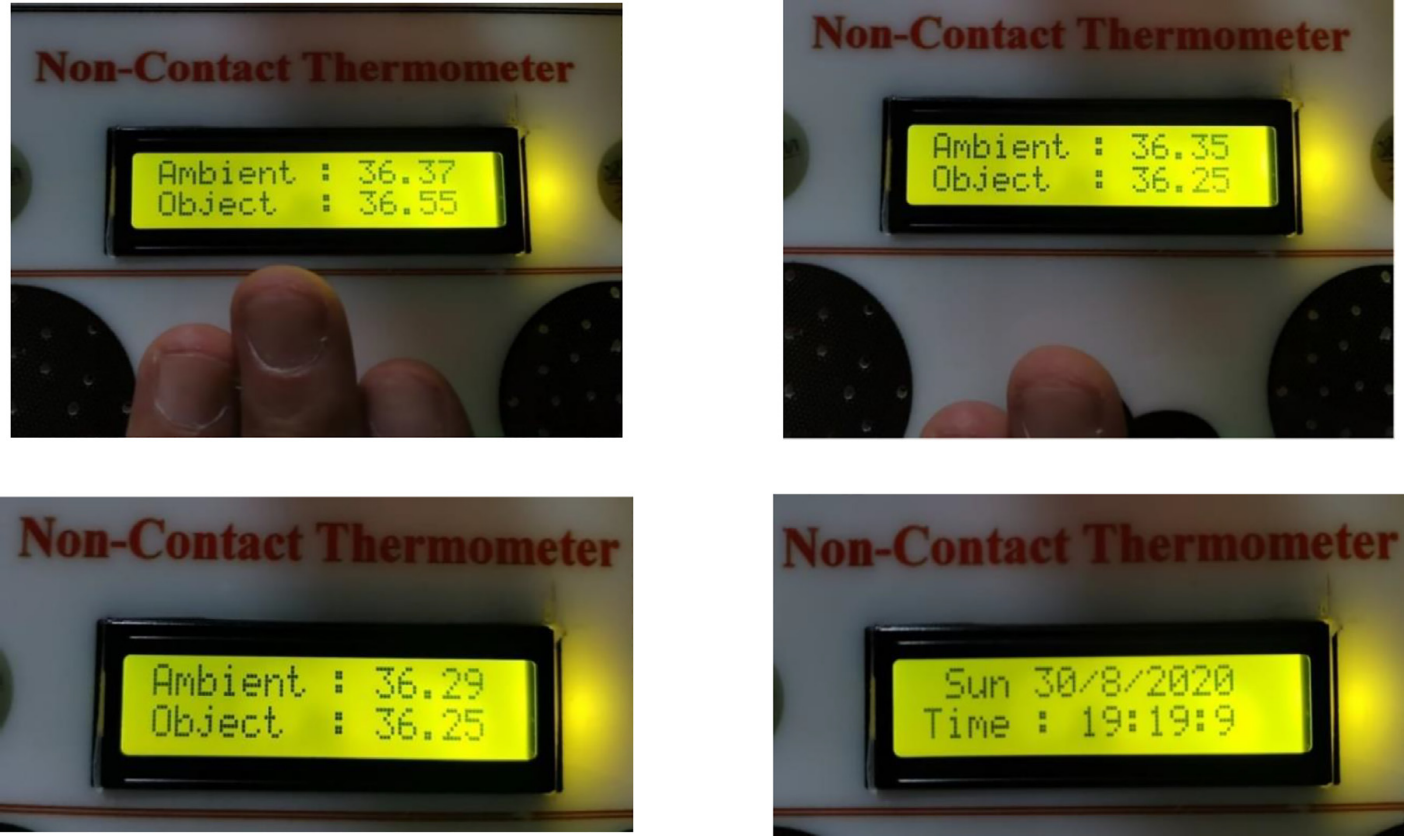
Different sets of temperature measurements for a group of people.

After the device was designed and developed as a prototype in its final form (as seen in Fig. 8), the temperatures of a group of people were taken with it, all readings were identical to those given by other devices (see Fig. 9)

**Fig. 10:**
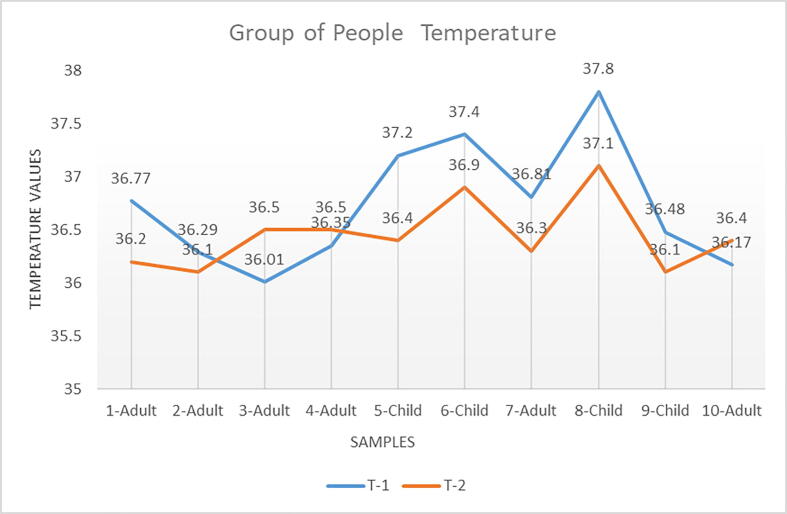
shows the comparison temperature chart between the results of our device with one of the devices on the market that serves the same purpose. The device used for comparison is present in the Fig. 11, this device is “Non-contact Forehead Thermometer Digital Infrared Body”. *Note*: T-1 The temperature of the developed device in this paper. T-2 The temperature of the commercial device compared with it (this device is presented in Fig. 11)

**Fig. 11:**
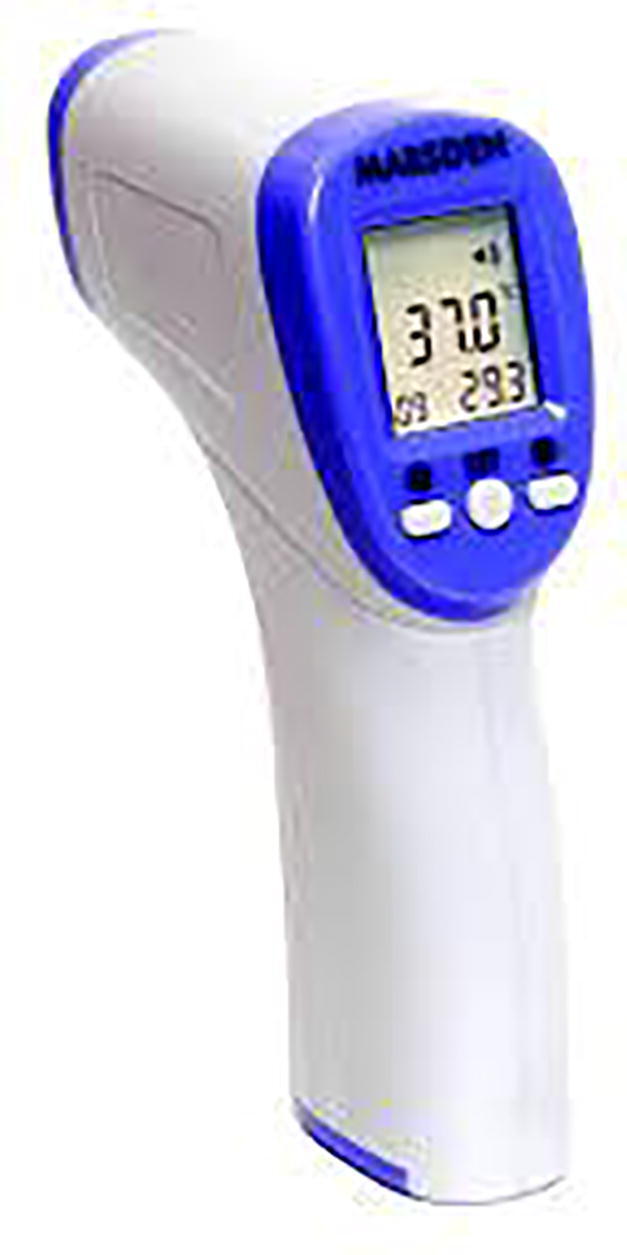
A model for one of the Non-Contact temperature measurement devices in the local market.

A set of readings of body temperature were taken for more than 100 people of different ages and different times. The results were very close. Regardless of the site of temperature measurement, the repeated measurements showed a large individual variability and that replicated measurements do not increase precision. The mean difference was >0.5 °C with a large individual variance when comparing the hand temperature with nasal, rectal, ear and axillary readings. In addition to that, the purpose of developing this device is for the initial detection of high temperature for people from a distance without having direct contact so that no infection is transmitted.

### 5.7 Pearson correlation coefficient calculator

To calculate the intensity of a linear relationship between two variables, the Pearson correlation coefficient is used, where the value r = 1 implies a perfect positive correlation and the value r = -1 implies a perfect negative correlation. So, for instance, you might use this test to figure out whether the height and weight of people are associated (they would be - the taller individuals are, the heavier they are likely to be) [19]. Equation (1) present the Pearson correlation coefficient and the requirements below.

• Scale of measurement should be interval or ratio
• Variables should be approximately normally distributed
• The association should be linear
• There should be no outliers in the data

(1) $$r=\frac{\sum_{i}\left(T1i-M\left(T1\right)\right)\left(T2i-M\left(T2\right)\right)}{\sqrt{\sum_{i}{\left(T1i-M\left(T1\right)\right)}^{2}}\sqrt{\sum_{i}{\left(T2i-M\left(T2\right)\right)}^{2}}}$$

**Table:** 

***T1***	T-1 The temperature values of the developed device in this paper.
***T2***	T-2 The temperature Values of the commercial device
***M(T1)***	Mean of T1 Values
***M(T2)***	Mean of T2 Values
$\left(T1i-M\left(T1\right)\right)$: and $(T2i-M\left(T2\right)$***)***	Deviation scores
${\left(T1i-M\left(T1\right)\right)}^{2}$: and ${\left(T2i-M\left(T2\right)\right)}^{2}$	Deviation Squared
$\left(T1i-M\left(T1\right)\right)\left(T2i-M\left(T2\right)\right)$	Product of Deviation Scores

• **T1 Values**
  ∑ = 367.28
  Mean = 36.728
  ∑(T1 – M(T1))2 = SS(T1) = 3.055
• **T2 Values**
  ∑ = 364.5
  Mean = 36.45
  ∑(T2 – M(T2))2 = SS(T2) = 0.965
• **T1 and T2 Combined**
  N = 10
  ∑(T1 – M(T1))(T2 – M(T2)) = 1.166
• **r Calculation**
  r = 1.166 / √((3.055)* √ (0.965)) = 0.6791

Details of the results and calculation for Equation (1) are presented in Table 1 below, depending on T-1 and T-2 in Fig. 10

**Table 1:** Calculation Results of the Equation (1).

T-1	T-2	T1-M(T1)	T2-M(T2)	(T1-M(T1))^2^	(T2-M(T2))^2^	(T1-M(T1))*(T2-M(T2))
36.77	36.2	0.042	−0.25	0.002	0.062	−0.01
36.29	36.1	−0.438	−0.35	0.192	0.123	0.153
36.01	36.5	−0.718	0.05	0.516	0.002	−0.036
36.35	36.5	−0.378	0.05	0.143	0.002	−0.019
37.2	36.4	0.472	−0.05	0.223	0.003	−0.024
37.4	36.9	0.672	0.45	0.452	0.202	0.302
36.81	36.3	0.082	−0.15	0.007	0.023	−0.012
37.8	37.1	1.072	0.65	1.149	0.422	0.697
36.48	36.1	−0.248	−0.35	0.062	0.123	0.087
36.17	36.4	−0.558	−0.05	0.311	0.003	0.028
**∑ = 367.28**	**∑ = 364.5**			**Sum: 3.055**	**Sum: 0.965**	**Sum: 1.166**
**M(T1): 36.728**	**M(T2): 36.450**					

## 6 Operation instructions

The device is designed for ease of use and to take temperature readings without the need for professional intervention. A user needs only to position their hand above the prototype device meter at 5 cm to 15 cm distance; the infrared signal (IR) emitted from the thermometer helps the instrument to start the operation for measuring temperature.

**Fig. 12:**
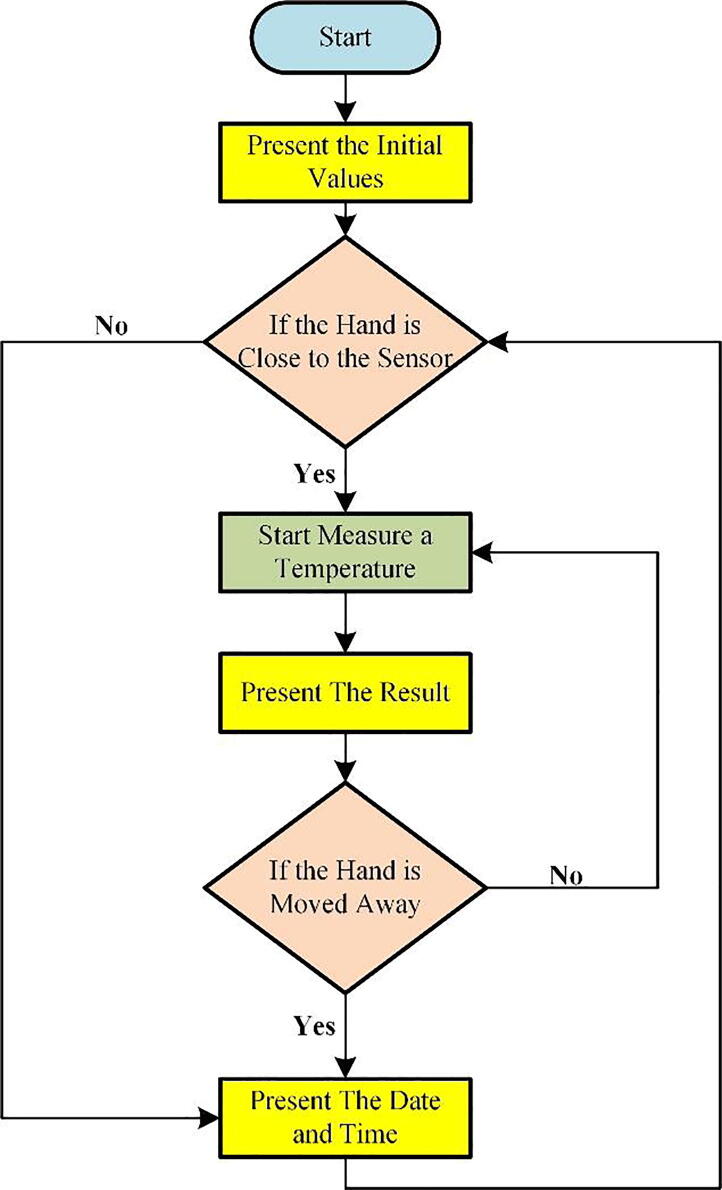
The Operating Instructions Flowchart.

The thermometer measures the thermal radiation emitted from the object to which it is directed and displays the temperature reading on the LCD screen. Depending on the temperature measured, the device either allow the user in (if the temperature is acceptable) or requests them to try again three times only, after that it will be reject to allow entering. The operation instruction is presented in the flowchart in Fig. 12.

## Declaration of Competing Interest

The authors declare that they have no known competing financial interests or personal relationships that could have appeared to influence the work reported in this paper.
